# Knee osteoarthritis alters peri-articular knee muscle strategies during gait

**DOI:** 10.1371/journal.pone.0262798

**Published:** 2022-01-20

**Authors:** Aseel Ghazwan, Chris Wilson, Cathy A. Holt, Gemma M. Whatling

**Affiliations:** 1 Cardiff School of Engineering, College of Physical Sciences and Engineering, Cardiff University, Cardiff, United Kingdom; 2 Biomechanics and Bioengineering Research Centre Versus Arthritis, Cardiff University, Cardiff, United Kingdom; 3 Biomedical Engineering Department, College of Engineering, Al-Nahrain University, Baghdad, Iraq; 4 University Hospital of Wales, Cardiff, United Kingdom; University of Illinois at Urbana-Champaign, UNITED STATES

## Abstract

The primary role of muscles is to move, and control joints. It is therefore important to understand how degenerative joint disease changes this role with the resulting effect on mechanical joint loading. Muscular control strategies can vary depending on strength and coordination which in turn influences joint control and loading. The purpose of this study was to investigate the variation in neuromuscular control mechanisms and joint biomechanics for three subject groups including those with: uni-compartmental knee osteoarthritis (OA), listed for high tibial osteotomy surgery (pre-HTO, n = 10); multi-compartmental knee OA listed for total knee replacement (pre-TKR, n = 9), and non-pathological knees (NP, n = 11). Lower limb kinematics and electromyography (EMG) data for subjects walking at self-selected speed, were input to an EMG-driven musculoskeletal knee model which was scaled and calibrated to each individual to estimate muscle forces. Compared to NP, the peak gastrocnemius muscle force reduced by 30% and 18% for pre-HTO and pre-TKR respectively, and the peak force estimated for hamstring muscle increased by 25% for pre-HTO. Higher quadriceps and hamstring forces suggest that co-contraction with the gastrocnemius could lead to higher joint contact forces. Combined with the excessive loading due to a high external knee adduction moment this may exacerbate joint destruction. An increased lateral muscle co-contraction reflects the progression from NP to uni-compartmental OA (pre-HTO). Pre-TKR patients adopt a different gait pattern to pre-HTO patients. Increased medial muscle co-activation could potentially differentiate between uni- or multi-compartmental OA.

## 1. Introduction

Mechanical factors play an important role in the development and progression of Osteoarthritis (OA) [1]. The knee joint is recognized as the most commonly affected joint [2] and about 4.71 million people have sought treatment for knee OA [3]. Estimates suggest that the number of people with knee OA will increase from 4.71 million in 2010, to 5.4 million in 2020, reaching 6.4 million by 2035 [3]. Typically, total knee joint loading is influenced by ground reaction, joint contact, muscle, and soft tissue forces. The net moment resulting from the ground reaction force is counterbalanced by the moments produced by muscles and contact forces. 60–80% of total intrinsic knee load is primarily produced by muscle forces [4, 5] and is distributed through the medial compartment of the tibio-femoral joint [6]. The pattern and magnitude of knee compressive forces are directly affected by the way that individuals activate their muscles. Therefore, assessments of muscle forces and joint moments are essential to fully understand altered loading mechanisms associated with the incidence and progression of OA.

Most gait studies have focussed their investigations on knee kinematics and kinetics [7–10], muscle activations [11–15], and muscle forces [16, 17]. In contrast, a limited number of studies have investigated all these variables together and related them to the compartmental involvement of knee OA [18–20].

External knee adduction moment (EKAM), suggested as a surrogate measure of knee OA severity [21, 22], has been used to evaluate joint loading, with peak EKAM providing a strong predictor of medial knee contact force [23]. However, being a net moment, it does not explicitly account for individual muscle activation patterns as well as muscle co-contractions.

Muscle activation features also play an important role in understanding the effects of longitudinal progression or interventions for knee OA. Hubley-Kozey et al. [24] showed that reliable EMG characteristics can be captured for patients with moderate medial compartment knee OA. Lloyd and Buchanan [15] investigated the activation strategies used by individuals to support adduction/abduction moments and the muscle loading patterns that result from these activation schemes during highly controlled isometric tasks. Wilson et al. [25] associated EMG patterns of the knee periarticular musculature with post-operative tibial implant migration. They found that a prolonged muscle activation pattern for both the lateral gastrocnemius and vastus medialis muscles, during the stance phase, was related to increased posterior migration of the tibial component. Moreover, higher muscle co-contractions have been linked to OA severity [14, 26–28], presumed to be linked with higher muscle forces [29, 30], to compensate for joint instability.

Measuring the forces applied to a joint and estimating how these forces are partitioned with respect to surrounding muscles, ligaments, and articular surfaces is fundamental to understanding joint function, injury, and disease. Muscle forces have been proposed as the primary determinants of joint contact forces [4, 31], with correctly predicted muscle forces assumed to result in sensible estimates of joint contact loads. However, to date, accurate measurement and prediction of individual muscle forces are still a major challenge.

Advances in musculoskeletal knee modelling and computation power have enabled researchers to generate gait simulations in efforts to estimate joint moments, muscle forces (e.g., [32, 33]) and subsequently joint loading, for patient populations that use altered neuromuscular activation patterns (e.g., [32–35]). Among the four prominent methods introduced to estimate muscle forces (EMG-driven model [36], Static optimization [37], computed muscle control [38] and DeVita model [39]), the EMG-driven model is preferred as it accounts for subject specific EMG patterns resulting in improved estimations of muscle forces.

Although biomechanical evaluations of people with OA are frequently performed to identify gait impairments; little attention has been paid to the provision of quantitative information regarding the function of individual muscles. Adouni and Shirazi-Adl [40] developed a gait-data driven musculoskeletal model of the lower extremity to estimate muscle forces and knee joint stresses-strains during the stance phase of the walking cycle in a subject with knee OA and a non-pathologic (NP) subject. The OA patient adopted reduced muscle forces through stance phase, except at mid stance, compared to the NP subject. However, Kumar et al. [41] demonstrated that OA patients had higher hamstring and gastrocnemius muscle forces at both loading response and mid-stance phases of the gait cycle.

This paper contributes to our current understanding of the underlying neuromuscular mechanisms during gait for patients with knee OA prior to HTO and TKR surgery. A question has been posed: Do muscle coordination strategies, adopted by subjects with knee OA, and associated changes in knee function reflect the level of involvement of compartmental knee OA?

## 2. Methods

Kinematic and kinetic data were collected in the Motion Analysis Laboratory (Cardiff School of Engineering) from thirty subjects, each gave their informed written consent prior to data collection, divided into three groups: eleven subjects with no knee pathology (NP), ten subjects with medial uni-compartmental knee OA and correctable varus deformity listed for high tibial osteotomy (pre-HTO) [42], and nine subjects with multi-compartmental knee OA listed for total knee replacement (pre-TKR). Ethical approval was granted from the Wales Research Ethics Committee 3 (10/MRE09/28) and Cardiff and Vale University Health Board.

Motion analysis was performed using nine 120Hz infra-red motion capture units (Qualisys, Gothenburg, Sweden). Qualisys Track Manager (QTM, Qualisys, Gothenburg, Sweden) was used to capture full body motion using reflective markers placed on the trunk, pelvis, and both the upper and lower limbs (modified Cleveland clinic marker set) [7, 43]. Four floor-embedded force platforms (Bertec Corporation) were used to capture the ground reaction force vectors with a sample rate of 1080 Hz. Motion data were processed using the Matlab Motion data elaboration Toolbox for NeuroMusculoSkeletal applications (MOtoNMS) [44].

Muscle electromyographic (EMG) data were collected bilaterally, using a Trigno™ Wireless EMG System (Delsys Incorporated, Natick, MA, USA), for seven muscles: rectus femoris, vastus lateralis, vastus medialis, biceps femoris, semitendinosus, gastrocnemius lateralis, and gastrocnemius medialis. The electrodes were placed longitudinally over the muscle bellies after standard preparation of the skin, according to SENIAM recommendations [45], involving shaving, exfoliation, cleaning of the skin and finally electrode gel was used to reduce the electrode–skin impedance [46].

Participants were asked to perform activities of daily living whilst muscle EMG, ground reaction force and three-dimensional movements were collected using the synchronized movement analysis system. Six trials of level gait at self-selected walking speeds, six trials of ascending/descending a four-step staircase [47], and two trials of standing/sitting were recorded for each subject. The stance phase was determined by the ground reaction force measured from heel strike to toe-off.

Recorded raw EMG data, through stance phase, were analysed in Matlab (version R2013a, Mathworks Inc.). The raw EMG signals were band-pass filtered, to remove the movement artefacts, by a Butterworth 4th order filter at (10_450) Hz, rectified and finally low-pass-filtered with a 4th order Butterworth filter at 6 Hz to create a linear envelope for each muscle. Then linear envelopes for each muscle were normalized to peak values obtained through activities of daily living, as recommended by [48]. Finally, the co-contraction index (CCI) was calculated through stance, by using Eq (1) developed by Rudolph et al. [49], for the following muscle sets: vastus lateralis- gastrocnemius lateralis (VLLG), vastus lateralis-lateral hamstring (VLLH), vastus medialis- gastrocnemius medialis (VMMG), and vastus medialis-medial hamstring (VMMH).

$$CCI=\sum_{i=1}^{100}\left[\frac{low\;EMG_{i}}{high\;EMG_{i}}\times \left(high\;EMG_{i}+low\;EMG_{i}\right)\right]/100$$ (1)

Gait biomechanics were determined using OpenSim v3.3 [50]. For each participant, the customized generic anatomic model was scaled to the participant’s anthropometry. The final anatomic model was then used to calculate joint angles, moments and musculo-tendon unit kinematics (lengths and moment arms) for walking trials using OpenSim inverse kinematics (IK), inverse dynamics (ID) and muscle analysis tools, respectively. Gait biomechanics and processed EMGs were then used to calibrate and execute an EMG-driven model, for each subject, to estimate muscle forces by using CEINMS [51]. CEINMS has already been mentioned in depth [51, 52], so it will only be addressed briefly here. The musculo-tendon unit parameters of each individual were adjusted, which is part of the CEINMS framework, i.e., optimizing the musculo-tendon unit parameters to minimize the least square differences between the expected joint moments of the model and the experimentally measured joint moments. Two walks were included in the experimental trials used in the calibration process. The calibration parameter and boundary conditions list is the same as that used in [51]. After calibration, the model worked as an open-loop predictive ways to predict muscle forces and joint moments based on muscle activation and kinematic model.

Muscle forces and joint moments (hip, knee, and ankle) were normalized to each stance phase of the gait cycle. Values at heel strike, peak weight acceptance and peak push off, along with co-contraction indices were averaged across six representative stance phases for each subject, and then averaged across subjects to obtain group means. Heel strike was defined as the value at 0% of the stance phase. Peak weight acceptance was defined as the first 15% of the stance phase. Peak push off was defined as the mean value between 5% on either side of the peak knee extension moment.

Patient-reported outcome measures (PROMs) including Oxford Knee Score [53], Knee Outcome Survey [54], Western Ontario and Mcmaster Universities Osteoarthritis Index [55], Pain Audit Collection System [56], and Knee Injury And Osteoarthritis Outcome Score [57] were completed to provide a subjective measure of how each patient perceived their knee function.

## 3. Statistical analysis

The Kolmogorov–Smirnov and Levene tests were used to assess the normality of distribution (*P* > 0.05) and the equality of variances, respectively. One way analysis of variance (ANOVA) model was tested for significant group differences for demographics, walking speed, and patient reported outcome measures (PROMS), where data was normally distributed and homogenous. All significant findings were post hoc tested using Tukey adjusted alpha level to determine pair-wise significant differences.

Kinematic and kinetic data, were compared across the three subject groups, were not normally distributed or homogeneous. Accordingly, a Kruskal Wallis test of nonparametric data was performed. Pairwise comparisons using the Dunn-Bonferroni approach were automatically produced for any dependent variables for which the Kruskal-Wallis test was significant. All statistical analyses were performed using SPSS (version 20, Chicago, IL).

## 4. Results and discussion

Demographics and PROMS differed across the three subject groups; summarized in Tables 1 and 2. The Oxford Knee Score, Knee Outcome Survey, Western Ontario and Mcmaster Universities Osteoarthritis Index and Pain Audit Collection System were higher for the pre-TKR compared with the pre-HTO subjects indicating more severe self-reported pain and stiffness and reduced function. Knee Injury And Osteoarthritis Outcome Score was lower for pre-TKR compared to pre-HTO subjects, where lower scores indicate extreme knee problems.

**Table 1:** Demographics and clinical data mean (SD) for NP, Pre-HTO, and Pre-TKR subjects.

Characteristics	NP	Pre-HTO	Pre-TKR	*P*	*P*	*P*
	n = 11	n = 10	n = 9	NP vs. HTO	NP vs. TKR	HTO vs. TKR
**Age, years**	32.9 (5.2)	50.3 (6.9)	66.6 (9.8)	<0.001†	<0.001†	<0.001†
**Weight, kg**	79.5 (12.6)	87.8 (14.9)	88.7 (20.3)			
**Height, m** ^2^	1.76 (0.04)	1.73 (0.09)	1.69 (0.08)			
**Walking speed, m/s**	1.31 (0.23)	1.066 (0.16)	0.81 (0.19)	0.027†	<0.001†	0.024†
**Static varus angles**		9.57 (4.27)				
**Medial compartment KL score**		Grade 2: n = 4 Grade 3: n = 5 Grade 4: n = 1	Grade 3: n = 2 Grade 4: n = 7			

NP = Non-pathologic subjects.

† Significant differences between groups (*P*<0.05).

KL = Kellgren and Lawrence (range 0–4 where stage 0 is assigned to a normal, healthy knee and stage 4 to severe knee OA).

**Table 2:** Patient reported outcome measures (PROMS) mean (SD) for Pre-HTO, and Pre-TKR subjects.

Characteristics	Pre-HTO	Pre-TKR	*P*
	n = 10	n = 9	
**OKS**	25.8 (10.0)	29.2 (9.5)	0.456
**KOS**	48.8 (15.8)	49.1 (13.9)	0.964
**WOMAC**	38.9 (23.2)	49.8 (21.5)	0.303
**PACS**	39.3 (25.0)	47.6 (24.1)	0.475
**KOOS Pain**	63.1 (24.7)	54.0 (22.9)	0.439
**KOOS Symptom**	65.0 (19.5)	53.6 (22.2)	0.266
**KOOS ADL**	69.4 (24.4)	59.6 (21.5)	0.383
**KOOS Sport/Rec**	41.1 (32.7)	18.8 (23.4)	0.124
**KOOS QOL**	43.1 (21.7)	32.8 (22.1)	0.336

OKS = Oxford Knee Score (range from 12 for least difficulties to 60 for most difficulties).

KOS = Knee Outcome Survey (scale from 0–100 where 100 indicates no disability).

WOMAC = Western Ontario and McMaster University Osteoarthritis Index (high scores indicating high degree of impairment).

PACS = Pain Audit Collection System (scale from 0–10, from least to most difficulty or severity).

KOOS = Knee injury and osteoarthritis outcome score (range 0–100, with zero representing extreme knee problems).

ADL = Activities of daily living.

QOL = Quality of life.

No significant differences, between pre-HTO and pre-TKR groups, were observed in PROMS suggested that these outcome measures were not the most sensitive methods to differentiate between uni- and multi-compartmental knee OA.

**Fig 1:**
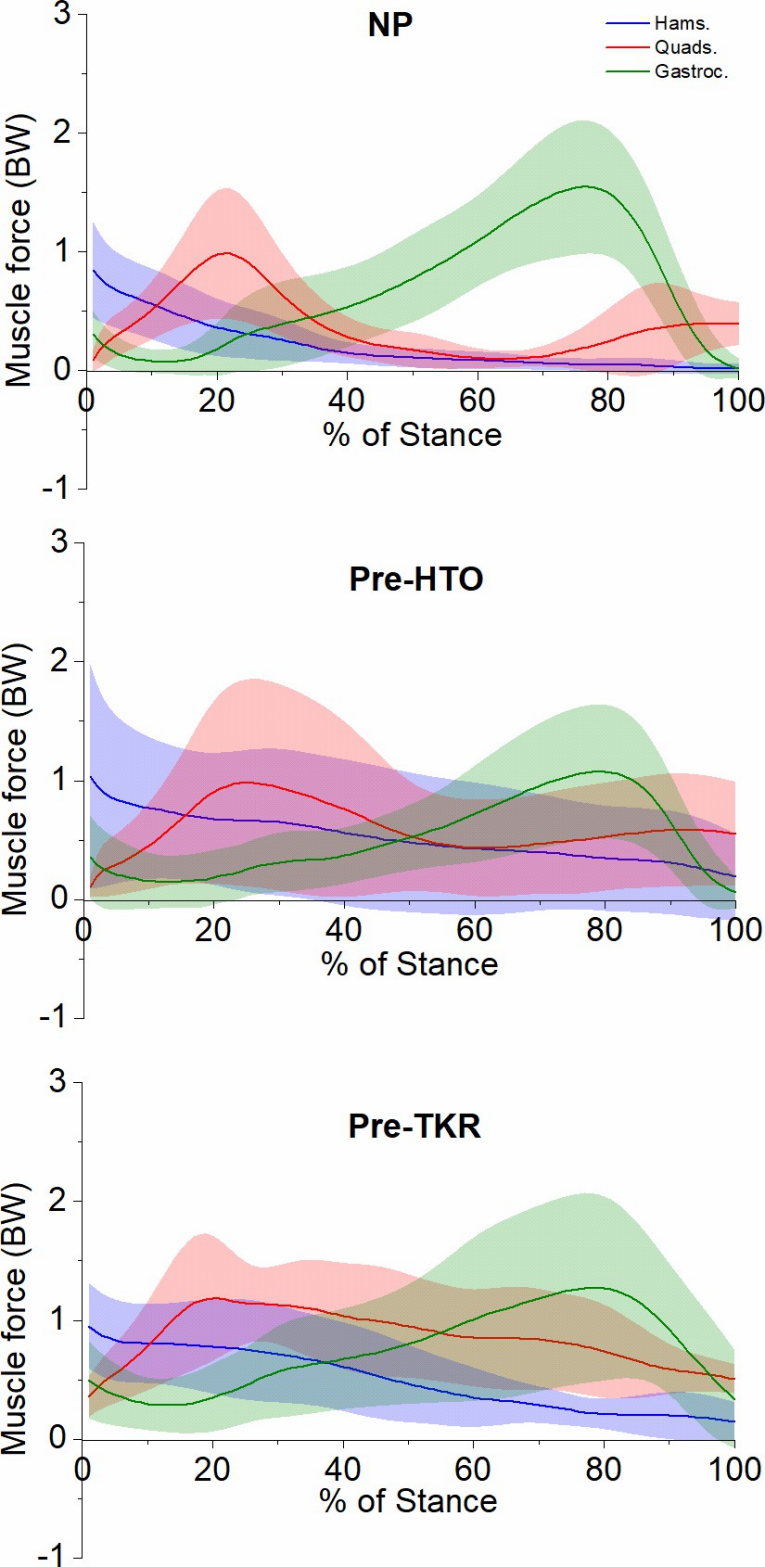
Muscle forces during stance-phase for NP, pre-HTO, and pre-TKR subjects. Values represent mean (SD). Quads = quadriceps force = ∑F_rectus femoris, vastus medialis_, _vastus lateralis, and vastus intermedius_. Hams = hamstring force = ∑F_semimembranosus, semitendinosus, and biceps femoris long and short head muscle forces_. Gastroc = gastrocnemius force = ∑F _medial and lateral gastrocnemius muscle forces_.

Group ensemble-averaged waveforms for quadriceps, hamstring and gastrocnemii muscle forces illustrate that peaks and patterns changed between NP, pre-HTO and pre-TKR groups (Fig 1). The sequence and timing of predicted muscle activity were consistent with a previous study [32], with gastrocnemius generating the largest forces about the knee at push off, for NP subjects. Pre-HTO subjects had elevated quadriceps and lateral hamstring activity compared to the NP subjects.

**Fig 2:**
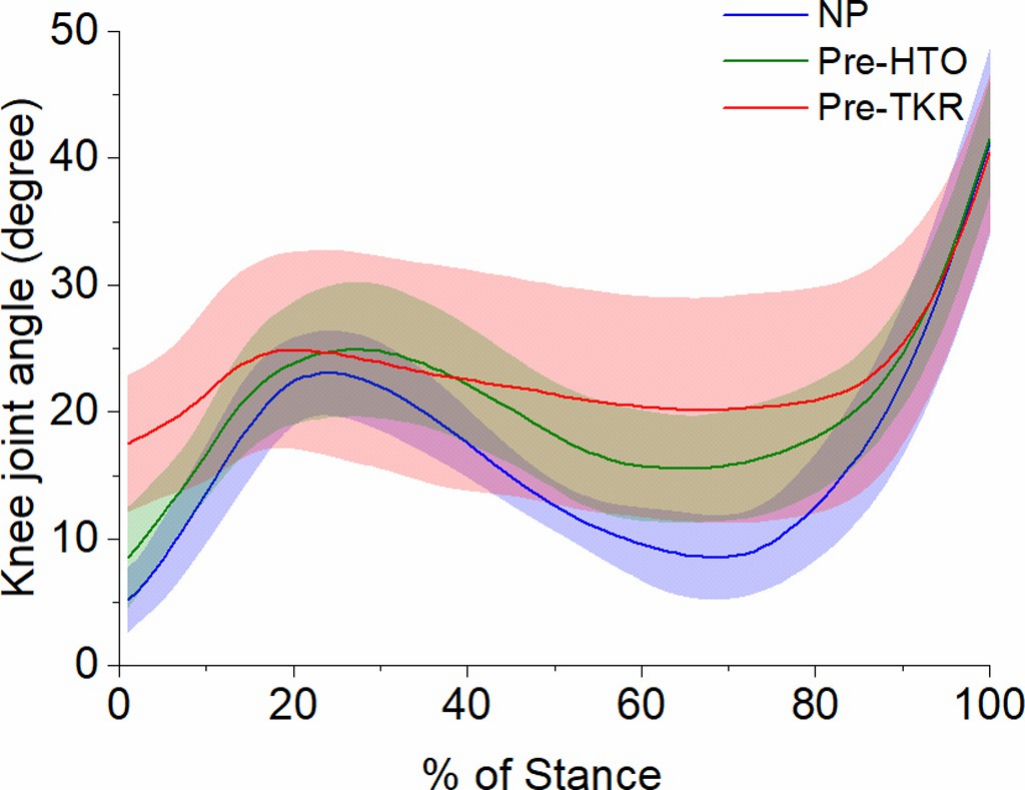
Group ensemble-averaged sagittal plane knee kinematic waveforms (knee flexion), during stance-phase for NP (blue), pre-HTO (green) and pre-TKR (red) subjects. Values represent mean (SD).

A reduced range of sagittal plane knee joint angle was observed for OA cohorts (pre-HTO and pre-TKR patients) as compared to NP, Fig 2.

**Fig 3:**
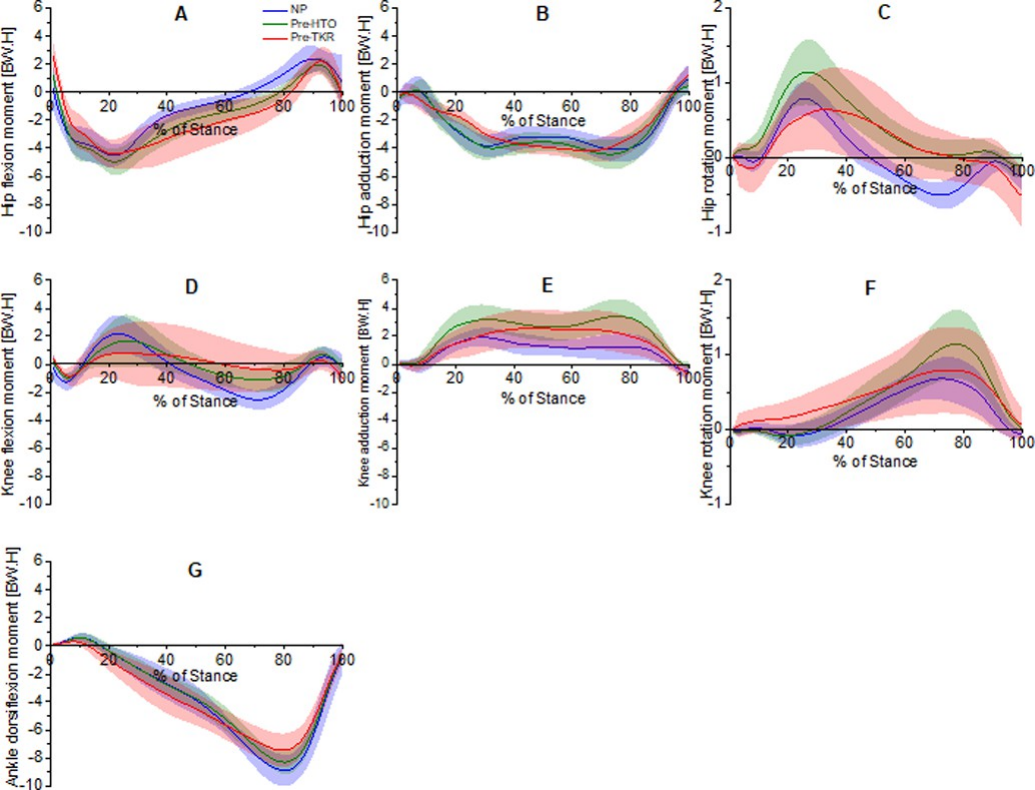
The external joint moment waveforms during stance-phase for NP (blue), pre-HTO (green) and pre-TKR (red) subjects: Hip flexion moment–**A**, Hip adduction moment–**B**, Hip rotation moment–**C**, Knee flexion moment–**D**, Knee adduction moment–**E**, knee rotation moment–**F**, Ankle flexion moment–**G**. Values represent mean (SD).

Hip, knee and ankle group ensemble-averaged moment curves (normalized to body weight and height (%BW*Ht)) for NP, pre-HTO and pre-TKR patients are shown in Fig 3. In agreement with [10], reduced sagittal plane joint moments were seen at the hip and knee joints (Fig 3A and 3D, respectively) for the knee OA group. Only the pre-TKR group had a reduced first peak hip adduction moment (Fig 3B). However, both knee OA cohorts had increased EKAM (Fig 3D). Pre-TKR subjects showed a trend of higher mid-stance EKAM (related to knee OA severity) with no clearly defined double peak (Fig 3D).

A summary of the individual muscle forces and joint moment is reported in Tables 3 and 4, respectively, during heel strike (HS), weight acceptance (WA), and push off (PO) phases of stance.

**Table 3:** Muscle forces for NP, pre-HTO and pre-TKR subjects at three different events: Heel strike (HS), weight acceptance (WA), and push off (PO) phases of stance.

	Variables	Group		
	Muscle Force (BW)	NP (n = 11)	Pre-HTO (n = 10)	Pre-TKR (n = 9)
**Heel Strike**	Biceps Femoris Long Head	1.59	0.255	0.208
	Biceps Femoris Short Head	0.114	0.274	0.153
	Semimembranosus	0.482	0.422	0.429
	Semitendinosus	0.087	0.063^c^	0.102
	Lateral Gastrocnemius	0.149	0.178	0.222
	Medial Gastrocnemius	0.154	0.3	0.351
	Rectus Femoris	0.017	0.022	0.053
	Vastus Intermedius	0.015^a^^,^^b^	0.036^c^	0.096
	Vastus Lateralis	0.018	0.044	0.086
	Vastus Medialis	0.03^b^	0.03^c^	0.106
**Weight Acceptance**	Biceps Femoris Long Head	0.16	0.261	0.224
	Biceps Femoris Short Head	0.119^a^	0.32	0.232
	Semimembranosus	0.484	0.422	0.445
	Semitendinosus	0.088	0.064^c^	0.107
	Lateral Gastrocnemius	0.15	0.178	0.24
	Medial Gastrocnemius	0.166	0.3	0.351
	Rectus Femoris	0.147	0.156	0.182
	Vastus Intermedius	0.216	0.201	0.272
	Vastus Lateralis	0.249	0.286	0.278
	Vastus Medialis	0.204	0.159	0.286
**Push off**	Biceps Femoris Long Head	0.006^a^^,^^b^	0.072	0.081
	Biceps Femoris Short Head	0.027^a^^,^^b^	0.172	0.166
	Semimembranosus	0.015	0.038	0.072
	Semitendinosus	0.009^a^^,^^b^	0.017	0.03
	Lateral Gastrocnemius	0.417	0.43	0.508
	Medial Gastrocnemius	1.04	0.626	0.763
	Rectus Femoris	0.059^a^^,^^b^	0.189	0.2
	Vastus Intermedius	0.026^a^^,^^b^	0.087	0.177
	Vastus Lateralis	0.029^a^^,^^b^	0.122	0.172
	Vastus Medialis	0.02^b^	0.06^c^	0.118

^a^ significant between NP and pre-HTO. (Kruskal–Wallis).

^b^ significant between NP and pre-TKR.

^c^ significant between pre-HTO and pre-TKR.

**Table 4:** knee, hip, and ankle kinetics for NP, pre-HTO and pre-TKR subjects at three different events: heel strike (HS), weight acceptance (WA), and push off (PO) phases of stance.

	Variables	Group		
	Joint kinetic [BW.H]	NP (n = 11)	Pre-HTO (n = 10)	Pre-TKR (n = 9)
**Heel Strike**	Knee flexion (+) /extension (−)	−0.23	0.22	0.42
	Knee adduction	0.08	0.05	0.04
	Knee internal (+) / external (−) rotation	0.01	−0.02	−0.02
	Hip flexion	0.30^b^	1.16^c^	2.58
	Hip adduction	−0.40	−0.30	−0.19
	Hip internal (+) / external rotation (−)	−0.01^a^	0.01^c^	0.03
	Ankle plantarflexion (+) / dorsiflexion (−)	0.09	0.06	0.14
**Weight Acceptance**	Knee flexion (+) /extension (−)	−1.36	−0.98	−0.87
	Knee adduction	1.01^a^	2.06	1.11
	Knee internal (+) / external (−) rotation	0.04	0.08^c^	0.14
	Hip flexion	4.03^a^	4.37^c^	3.87
	Hip adduction	0.12	0.16	−0.06
	Hip internal (+) / external rotation (−)	−0.06 ^a^	0.14	−0.25
	Ankle plantarflexion (+) / dorsiflexion (−)	0.62	0.58	0.40
**Push off**	Knee flexion (+) /extension (−)	−2.46^a^^,^^b^	−1.09	−0.40
	Knee adduction	1.21^a^	3.29	2.09
	Knee internal (+) / external (−) rotation	0.67	1.04	0.78
	Hip flexion	0.30^a^	−0.76	−0.70
	Hip adduction	−3.97	−4.37	−3.42
	Hip internal (+) /external rotation (−)	−0.49^a^^,^^b^	0.05	−0.01
	Ankle plantarflexion (+) / dorsiflexion (−)	−7.86	−7.28	−7.34

^a^ significant between NP and pre-HTO.

^b^ significant between NP and pre-TKR.

^c^ significant between pre-HTO and pre-TKR.

**Fig 4:**
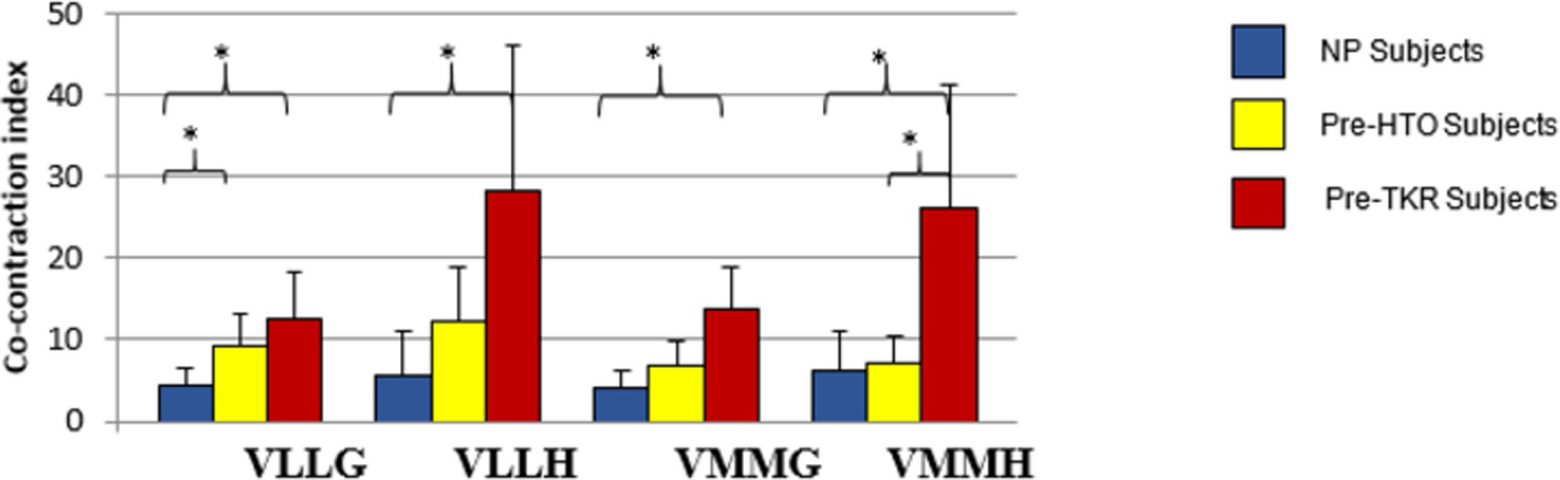
Co-contraction index during stance-phase for NP subjects–n = 11, pre-HTO subjects–n = 10, pre-TKR subjects–n = 9. Values represent mean (SD), (*) means the difference is significant (*P*< 0.05).

Fig 4 shows the CCI for NP, pre-HTO, and pre-TKR subjects. As expected, patients with knee OA have a higher CCI compared to NP subjects. Some evidence of co-contraction between the quadriceps and hamstrings muscles was found, in particular, for the VLLH in the pre-TKR compared to the NP subjects, which may contribute to a reduction in medial knee contact force.

This study has shown that neuromuscular control and biomechanics differ between non-pathological knees and those with OA. These differences are magnified as the level of compartmental involvement increases. The results have demonstrated that, in line with previous research findings [58], knee OA is a multifactorial disease process that involves many interrelated factors that interact to produce biomechanical changes throughout the disease process. In agreement with literature [10], a reduced range of motion for the sagittal plane knee joint angle was observed for knee OA cohorts as compared to NP, Fig 2. In principle, muscle control and external moments are the key to stance stability. Throughout stance, muscles contract when body alignment creates a moment that is antagonistic to weight bearing stability of the limb and trunk, and the amount of contraction is proportional to the magnitude of the demand torque that must be restrained.

Knee OA is associated with a reduction in gastrocnemius muscle force, whereas quadriceps and hamstring muscles play a significant role in controlling the knee joint. Subjects with knee OA had reduced gastrocnemius muscle forces compared to NP subjects by 30% and 18% for pre-HTO and pre-TKR, respectively. This could happen due to a reduction in knee extension moment (Fig 3D) for these groups; i.e, the role of a plantar flexor–knee extension couple. A plantar flexor–knee extension couple, which was addressed by [59, 60], plays a key role in knee control during gait. According to this phenomenon, under load, the planter flexors, i.e., gastrocnemius, are capable of acting on the tibiofemoral joint to extend the knee by holding back the tibia. Brunner and Rutz [61] suggested that the knee extensors (quadriceps) can control only the first phase of knee extension, during the response to loading. The second phase is controlled by the planter flexors. During this time interval, both knee OA groups had a reduced gastrocnemius muscle force compared to NP. In line with this finding, the results of this study have shown that patients with knee OA had reduced knee extension moments during late stance. Therefore, less gastrocnemius muscle force was developed in the knee OA (compared to NP group), to control the knee extension moment during late stance. The gastrocnemius is a biarticular muscle, crossing over the knee and ankle joints. Under load, the activity of the gastrocnemius can be adjusted according to this arrangement. Due to the lever arm situation at the knee under load, the gastrocnemius muscle is a powerful knee flexor when the knee is flexed. However, if its strength is needed at the ankle, simultaneous contraction of the vasti locks the knee and shifts the gastrocnemius’ strength to the ankle [62], i.e., the net effect is to extend the knee.

Our finding of increased quadriceps and hamstrings muscle forces in the knee OA groups is supported by an earlier study [63], where these muscle groups appear to be capable of supporting up to 100% of the applied adduction/abduction moment, where it is high in knee OA patients (Fig 3E), because of their abduction and/or adduction moment arm. The quadriceps play a major role in controlling the knee, as shortly after heel strike it acts to prevent excessive or rapid knee flexion. knee OA subjects used substantially higher quadriceps and hamstring muscle force than NP subjects, presumably in an attempt to support the joint against an excessive knee adduction moment, Fig 3E. The peak force estimated for hamstring muscle increased by 25%, for pre-HTO compared to NP.

In the NP stance phase, there are at least three crucial sub-phases. First is the **initial heel contact (HS)** when the line of action of the ground reaction force (GRF) passes posterior to the ankle centre, producing a plantar-flexion moment at the ankle. This moment is countered by the activation of ankle dorsiflexor muscles; this was the case for all groups. Considering the importance of leg stability during this phase, compensations aimed at extending the knee include premature activity of the gastrocnemius [62], avoiding knee flexion during loading response, with co-contraction of the knee extensors and hamstring muscles during knee flexion. Pre- TKR cohort shows a higher activity of the gastrocnemius compared to the other groups, Fig 1. At the knee joint, the ground reaction force passes anterior to the knee axis creating an extension moment, which is eccentrically controlled by the hamstring muscles to avoid hyper-extension of the knee and slow the forward movement of the leg. The results of this study show that at initial HS, there is an excessive knee flexion for the knee OA groups compared to the NP group, (18±5, 9±4, 5±3 degrees for Pre-TKR, Pre-HTO, and NP, respectively), leading to the GRF passing posterior to knee centre of rotation, creating a flexion moment rather than extension moment at the knee, (0.22, 0.42) %BW*H for pre-HTO and pre-TKR, respectively, Fig 3D. The GRF passes anterior to hip rotation centre creating a flexion moment, where the Pre- TKR cohort shows a significantly higher flexion moment compared to the pre-HTO and NP groups.

Second, is the **weight acceptance (WA) phase** where the ankle dorsi-flexors eccentrically contract towards foot flat. The knee extensors contract, to correct the position of the knee before it accepts further loading into single leg stance. Peak quadriceps action occurs during loading response, when the GRF vector is behind the joint axis. As the vector moves forward of the knee axis, an extension moment is created at the knee, and this muscle group relaxes. The gastrocnemius muscle group contracts to control knee extension at this stage, due to the plantar flexor–knee extension couple. Higher muscle forces occur during WA when the EKAM is high and the knee extends in single limb support. In this study the knee OA subjects (pre-HTO and pre-TKR), used substantially higher lateral hamstring and vastus lateralis muscle forces during WA phase compared to NP (Table 3). Further still, these were higher for pre-HTO subjects than for pre-TKR subjects. This finding further supports the notion that selective activation of lateral muscles is likely a pain and symptom management strategy in this population to unload the medial compartment of the knee.

Third, is the **push-off (PO) phase**, where the leg is accelerated forward by the gastrocnemius creating a rapid ankle plantar flexion to push off and with associated activity at the hip flexor (rectus femoris). During this phase, generally for NP gait, there is a maximal knee extension moment, a second peak knee adduction moment, and a peak dorsiflexion moment. In this study both knee OA groups had reduced the terminal knee extension moments (P<0.05) and ankle dorsiflexion moment (Fig 3G), compared to NP. This seems to be partially a compensatory action for the poorer physical capacity and the subjective knee pain experienced by this population. This, in addition to a reduced gastrocnemius force (Fig 1B and 1C and Table 3), implies that subjects with knee OA inadvertently used greater hip flexors (rectus femoris) muscle force to propel the body forward, i.e., 0.2, 0.18, and 0.06 (BW) for pre-TKR, pre-HTO and NP subjects, respectively. When comparing quadriceps muscle forces; vastus lateralis and vastus inter-medialis produced higher force in the pre-TKR subjects than NP (*P*<0.05) and pre-HTO subjects, and in the pre-HTO subjects compared to the NP subjects (*P*<0.05).

In the frontal plane, the hip adduction moment is generally found to be high in knee OA patients compared to NP. However, the pre-TKR cohort exhibited a different adduction pattern compared to the NP and pre-HTO cohorts. In both the NP and pre-HTO subjects, frontal plane moments had two distinct peaks. The first peak was higher for the pre-HTO compared to the NP group. This is attributed to altered alignment of the tibiofemoral joint (knee varus), which would create a valgus alignment at the hip.

The same trend was seen in the EKAM, where a significant increase (*P*<0.05) was observed for the pre-HTO subject group (2.06, 3.29) %BW*H compared to the NP (1.01, 1.21) %BW*H at weight acceptance and push-off, respectively. A remarkable increase in the first peak of EKAM for NP, in comparison to the second peak, was mainly due to the increase in the quadriceps force resisting the external knee flexion moment during this time interval. In contrast, the moderate increase of the second peak in the pre-HTO cohort, in comparison to the first peak, was due to the increase in both quadriceps and hamstring muscle forces during this time interval as compared to NP. The double peak could not be identified in pre-TKR patients.

In the transverse plane, the hip is significantly more internally rotated for the pre-HTO subjects group compared to the NP subject group (*P*<0.05), during weight acceptance and push off.

Higher symmetrical quadriceps and hamstring forces suggest that their co-contraction with the gastrocnemius muscles could lead to higher joint contact forces that, combined with the excessive loading due to high EKAM, would exacerbate joint destruction. Nonetheless, the knee OA groups used greater vastus lateralis muscle force compared to the NP subjects, which may imply that people with knee OA inadvertently increase the activity of vastus lateralis as a compensatory strategy to reduce medial joint compression and subsequent pain. For the knee OA subject groups, a reduced dorsiflexion moment and lower gastrocnemius co-contraction suggest that gastrocnemius overload may not be the primary cause of joint degeneration. If this is the case; attention should be focussed on the counterbalancing role of the quadriceps and hamstrings during dynamic movements to control knee biomechanics.

Pre-TKR patients (Fig 4), have significantly (*P*<0.05) higher CCI compared to NP subjects. Interestingly, this study shows that gastrocnemii indices are doubled in the pre-HTO group as compared to NP subjects. For the pre-TKR group, in addition to the gastrocnemii role that is associated with a two-fold increase in the CCI, the hamstring muscles also have the potential to control the knee joint. The hamstring indices increased by almost four-fold on the lateral side and three-fold on the medial side, as compared to NP individuals. There was also a significant increase in VLLH for the pre- TKR group, which may contribute to a reduction in the medial contact force. The lateral indices indicated an almost two-fold increase (*P*<0.05), for the pre-HTO group compared to NP subjects. Therefore, increased lateral muscle co-contraction reflects the non -pathological knee’s progression to uni-compartmental OA with varus deformity. No significant differences were found between pre-HTO and pre-TKR lateral indices.

Significant difference (*P*<0.05) was identified for the medial side index, VMMH, between the two OA groups, Fig 4. In agreement with [6], patients with uni-compartmental OA and varus deformity (pre-HTO) have increased lateral CCIs, which may help to unload the medial compartment. This increase is also evident for multi-compartmental OA (pre-TKR). Since increased medial muscle co-contraction has the potential to differentiate between uni- compartment and multi-compartment OA (Pre-HTO and pre-TKR patients), this could inform treatment management.

The results of this study should be viewed within the context of some limitations. The small sample size potentially lowers statistical power. However, a comparison of the joint angles, moments, and muscle activity from our simulation with the results from previous studies with larger populations demonstrated that our subjects displayed gait patterns that were typical. In addition, EMGs were recorded from just seven muscles bilaterally for use in the model to represent 10 muscles crossing the knee joint; some muscles in the model represented a combination of multiple muscles with similar functions, and this may affect the evaluation of magnitude for knee muscle forces. Finally, while musculoskeletal model parameters are adjusted for each individual, the model does not account for musculoskeletal size variations and alterations in muscle architecture. Variables such as maximum isometric force, optimum fibre length, and tendon slack length were modified to account for each subject’s weakened musculature.

## 5. Conclusion

Knee OA is associated with a reduction in gastrocnemius muscle force, whereas quadriceps and hamstring muscles play a significant role in controlling the knee joint, with altered coordination and increased forces with increasing age and involvement of knee compartment.

Moreover, pre-TKR patients adopt a gait pattern that differs from that of pre-HTO patients, attempting to unload the affected joint structures during walking, possibly by changing muscle coordination as well as moments at the adjacent ankle and/or hip. The increase of the lateral muscle co-contraction reflects the progression from NP to uni-compartmental OA and varus deformity (pre-HTO). Whereas, the increase of medial muscle co-activation could potentially differentiate between uni-compartmental or multi-compartmental OA.
